# Qualitative research

**DOI:** 10.31744/einstein_journal/2020ED5532

**Published:** 2020-03-18

**Authors:** Jacyr Pasternak

**Affiliations:** 1 Hospital Israelita Albert Einstein São PauloSP Brazil Hospital Israelita Albert Einstein, São Paulo, SP, Brazil.

Qualitative research articles are different from the usual research publications in medicine and biological sciences,^(1)^ given hypotheses are raised after data collection. The aim is to gain insights into and give voice to patients’ or persons’ experiences. The hypothesis can be altered along the way, which is impossible in most studies in Medicine. Surveys are often used in quantitative studies and can be combined with qualitative methods, such as in-depth interviews with small samples. Qualitative research is mostly based on face-to-face, telephone or remote (online video) interviews. The value of data obtained reflects the quality of the interviewer, should multiple interviewers be involved, inter-rater variability may adversely affect data interpretation.^(2)^ As in psychological group therapy, holding focus groups may help mitigating this problem.

Qualitative studies are common in Nursing and Social Sciences research, as well as in Anthropology. However, they are not that common in medical research, where they often precede quantitative studies. In other words, qualitative studies – often not published – can lead to quantitative investigations that are publishable.

There are good reviews on evaluation of qualitative methods, and I would recommend the excellent publication of the Swedish Agency for Health Technology Assessment and Assessment of Social Services and the one presented at the Washington symposium, both addressing the same topic.^(3,4)^

Many investigators and journals^(2)^ have restrictions regarding publication of qualitative research papers based on a scientific point of view. The lack of hypotheses and the fact the central research question can be altered over the course of the study make us argue about the validity of qualitative data as science. For many people, qualitative studies are more similar to a form of art, which can be expressed as romances or novels. This does not decry the value of some studies. The writings about the Sakhalin penal colony in Russia, by Chekhov’s, or the monumental work War and Peace by Lev Tolstoy, and to cite some Brazilian authors, the works by Euclides da Cunha (*Os sertões*) and Guimarães Rosa (*Grande Sertão: Veredas*) could be conceived as qualitative research.

## References

[1] 1. United Kingdom. Public Health England. Evaluation in health and wellbeing: overview. Guidance Evaluation methods [Internet]. United Kingdom: Published 7 August 2018 [cited 2019 Dec 11]. Available from: https://www.gov.uk/government/publications/evaluation-in-health-and-well-being-overview/evaluation-methods

[2] 2. Giacomini MK, Cook DJ. Users’ guides to the medical literature: XXIII. Qualitative research in health care A. Are the results of the study valid? Evidence-Based Medicine Working Group. JAMA. 2000;284(3):357-62. 10.1001/jama.284.3.357 10891968

[3] 3. Board on Global Health; Institute of Medicine. Evaluation Design for Complex Global Initiatives: Workshop Summary. 6 Applying Qualitative Methods to Evaluation on a Large Scale [Internet]. Washington (DC): National Academies Press (US); 2014 Jun 23 [cited 2019 Dec 11]. Available from: https://www.ncbi.nlm.nih.gov/books/NBK223145/ 25057695

[4] 4. Swedish Agency for Health Technology Assessment and Assessment of Social Services (SBU). Evaluation and synthesis of studies using qualitative methods of analysis [Internet]. Stockholm: SBU; 2016 [cited 2019 Dec 11]. Available from: https://www.sbu.se/globalassets/ebm/metodbok/sbuhandbook_qualitativemethodsofanalysis.pdf

