# Supplementary material for: Qualitative research
Source: Einstein (Sao Paulo). 2020 Mar 18;18:eED5532. doi: 10.31744/einstein_journal/2020ED5532 (PMC7087455; doi:10.31744/einstein_journal/2020ED5532)
Supplement: Translation

# Translation: Pesquisas qualitativas

**Authors:** Jacyr Pasternak

**Affiliations:** 1 Brasil Hospital Israelita Albert Einstein, São Paulo, SP, Brasil.

Artigos baseados em pesquisas qualitativas diferem daqueles comumente publicados nas áreas de Medicina e Ciências Biológicas,^(1)^ por envolverem formulação de hipóteses após a coleta de dados. A intenção é aguçar percepções e dar voz à experiência de pacientes e de demais indivíduos. A hipótese pode ser alterada ao longo da pesquisa, o que é impossível na maioria dos estudos em Medicina. O uso de enquetes é comum em estudos quantitativos. Entretanto, estas podem ser combinadas com métodos qualitativos, como entrevistas em profundidade, com amostras pequenas. A maioria das pesquisas qualitativas se baseia em entrevistas presenciais, telefônicas ou remotas (conduzidas por meio de vídeo). O valor dos dados obtidos reflete a qualidade do entrevistador e, na presença de múltiplos entrevistadores, a variabilidade entre os mesmos pode dificultar sua interpretação.^(2)^ Assim como na psicoterapia em grupo, a criação de grupos focais (*focus groups*) pode ajudar a mitigar o problema.

Estudos qualitativos, comuns nas áreas de Enfermagem e Ciências Sociais, assim como na Antropologia, são menos comuns na área médica, na qual costumam preceder estudos quantitativos. Em outras palavras, estudos qualitativos – em geral, não publicados – podem dar origem a estudos quantitativos dignos de publicação.

Existem boas revisões abordando a avaliação de métodos qualitativos de pesquisa, e minhas recomendações pessoais seriam a excelente publicação da *Swedish Agency for Health Technology Assessment and Assessment of Social Services* e aquela apresentada no simpósio realizado em Washington, ambas abordando o mesmo tema.^(3,4)^

Muitos pesquisadores e periódicos^(2)^ têm restrições quanto à publicação de artigos sobre pesquisas qualitativas, do ponto de vista científico. A ausência de hipóteses e o fato de o objetivo da investigação poder ser alterado ao longo do estudo nos levam a questionar a validade de dados qualitativos enquanto ciência. Para muitos, os estudos qualitativos se assemelham mais à arte, algo que poderia ser escrito na forma de romance ou novela, como os escritos de Chekhov sobre a colônia penal russa de Sacalina, ou os de Lev Tolstoy, em sua obra monumental *Guerra e Paz*. E, para citar alguns autores brasileiros, as obras de Euclides da Cunha (Os sertões) e Guimarães Rosa (Grande Sertão: Veredas) poderiam ser consideradas pesquisas qualitativas.
